# Supplementary material for: Cytokine Deficiencies in Patients with Long-COVID
Source: J Clin Cell Immunol. Author manuscript; Available in PMC 2023 Feb 2. (PMC9894377)
Supplement: Suppl. Table 1 [file NIHMS1854241-supplement-Suppl__Table_1.pdf]

Supplemental Table 1. Comparison of publications depicting cytokine fluctuation in ME/CFS and our long-COVID cytokine results.

Cytokine	Higher in ME/CFS	Lower in ME/CFS	No Change	Total Studies	Long-COVID
**Pro-inflammatory**					
IL-1b	**7 (28%)** [1-7]	0	18 (72%) [8-25]	25	**NC**
TNFa	**4 (15%)** [1, 2, 5, 21]	0	22 (85%) [4, 6-20, 22, 24-28]	26	**NC**
IL-6	**6 (24%)** [1, 4, 6, 7, 11, 29]	2 (8%) [2, 9]	17 (68%) [8, 10, 12-16, 18, 19, 21, 23, 24, 26, 30-33]	25	**Down**
**Anti-inflammatory**					
IL-10	**2 (13%)** [1, 2]	1 (6%) [9]	12 (81%) [4, 6-8, 11-16, 18, 28]	15	**NC**
IL-13	**1 (16%)** [1]	0	5 (84%) [6-9, 13-15]	6	**Down**
**Th1**					
IL-2	**2 (14%)** [11, 13]	0	12 (86%) [1, 4, 7-9, 13-15, 18, 21, 25, 34]	14	**Down**
IL-12	**3 (30%)** [1, 7, 11]	0	7 (70%) [6, 8, 9, 13-15, 18]	10	**NC**
IFNγ	**2 (13%)** [1, 11]	1 (6%) [2]	12 (81%) [6-10, 13-15, 22, 23, 25, 28]	15	**Down to 0**
**Th2**					
IL-5	*1 (14%)* [7]	*1 (14%) [1]*	5 (72%) [8, 9, 11, 13, 14]	7	**NC**
IL-4	**2 (18%)** [1, 7]	0	9 (82%) [8, 9, 11-16, 21]	11	**Down**
**Th17**					
IL-17	0	**2 (33%) [1, 9]**	4 (66%) [7, 8, 13, 14]	6	**Down**
**NK-cell attractant**					
IL-8	*3 (23%)* [2, 6, 14]	*3 (23%)* [1, 7, 9]	7 (54%) [8, 11-13, 15, 16, 29]	13	**Down to 0**

Adapted from Blundell *et al.* 2015[35]. There is no agreement regarding changes in cytokine levels associated with ME/CFS. Although studies that do report changes tend to report cytokines as being upregulated in ME/CFS as compared to our long-COVID cytokine data showing significant decreases in cytokine levels.

Supplemental References:

[1] S.F. Khaiboullina, K.L. DeMeirleir, S. Rawat, G.S. Berk, R.S. Gaynor-Berk, T. Mijatovic, N. Blatt, A.A. Rizvanov, S.G. Young, V.C. Lombardi, Cytokine expression provides clues to the pathophysiology of Gulf War illness and myalgic encephalomyelitis, Cytokine 72(1) (2015) 1-8.
[2] D. Neu, O. Mairesse, X. Montana, M. Gilson, F. Corazza, N. Lefevre, P. Linkowski, O. Le Bon, P. Verbanck, Dimensions of pure chronic fatigue: psychophysical, cognitive and biological correlates in the chronic fatigue syndrome, Eur J Appl Physiol 114(9) (2014) 1841-51.
[3] M. Maes, K. Ringel, M. Kubera, G. Anderson, G. Morris, P. Galecki, M. Geffard, In myalgic encephalomyelitis/chronic fatigue syndrome, increased autoimmune activity against 5-HT is associated with immuno-inflammatory pathways and bacterial translocation, J Affect Disord 150(2) (2013) 223-30.
[4] E.G. Lattie, M.H. Antoni, M.A. Fletcher, F. Penedo, S. Czaja, C. Lopez, D. Perdomo, A. Sala, S. Nair, S.H. Fu, N. Klimas, Stress management skills, neuroimmune processes and fatigue levels in persons with chronic fatigue syndrome, Brain Behav Immun 26(6) (2012) 849-58.
[5] M. Maes, F.N. Twisk, M. Kubera, K. Ringel, Evidence for inflammation and activation of cell-mediated immunity in Myalgic Encephalomyelitis/Chronic Fatigue Syndrome (ME/CFS): increased interleukin-1, tumor necrosis factor-alpha, PMN-elastase, lysozyme and neopterin, J Affect Disord 136(3) (2012) 933-9.
[6] P. Scully, D.P. McKernan, J. Keohane, D. Groeger, F. Shanahan, T.G. Dinan, E.M. Quigley, Plasma Cytokine Profiles in Females With Irritable Bowel Syndrome and Extra-Intestinal Co-Morbidity, Official journal of the American College of Gastroenterology | ACG 105(10) (2010) 2235-2243.
[7] M.A. Fletcher, X.R. Zeng, Z. Barnes, S. Levis, N.G. Klimas, Plasma cytokines in women with chronic fatigue syndrome, J Transl Med 7 (2009) 96.
[8] V.B. Wyller, O. Sorensen, D. Sulheim, E. Fagermoen, T. Ueland, T.E. Mollnes, Plasma cytokine expression in adolescent chronic fatigue syndrome, Brain Behav Immun 46 (2015) 80-6.
[9] J.G.M. Mady Hornig, Nancy G. Klimas, Susan Levine, Donna Felsenstein, Lucinda Bateman, Daniel L. Peterson, C. Gunnar Gottschalk, Andrew F. Schultz, Xiaoyu Che, Meredith L. Eddy, Anthony L. Komaroff, W. Ian Lipkin, Distinct plasma immune signatures in ME/CFS are present early in the course of illness, Sci Adv (2015).
[10] Y. Nakatomi, K. Mizuno, A. Ishii, Y. Wada, M. Tanaka, S. Tazawa, K. Onoe, S. Fukuda, J. Kawabe, K. Takahashi, Y. Kataoka, S. Shiomi, K. Yamaguti, M. Inaba, H. Kuratsune, Y. Watanabe, Neuroinflammation in Patients with Chronic Fatigue Syndrome/Myalgic Encephalomyelitis: An (1)(1)C-(R)-PK11195 PET Study, J Nucl Med 55(6) (2014) 945-50.
[11] M.N. Garcia, A.M. Hause, C.M. Walker, J.S. Orange, R. Hasbun, K.O. Murray, Evaluation of prolonged fatigue post-West Nile virus infection and association of fatigue with elevated antiviral and proinflammatory cytokines, Viral immunology 27(7) (2014) 327-33.

[12] T. Nakamura, S. Schwander, R. Donnelly, D.B. Cook, F. Ortega, F. Togo, Y. Yamamoto, N.S. Cherniack, M. Klapholz, D. Rapoport, B.H. Natelson, Exercise and sleep deprivation do not change cytokine expression levels in patients with chronic fatigue syndrome, Clinical and vaccine immunology : CVI 20(11) (2013) 1736-42.
[13] G.B. Anne Liese Smylie , Henrique Fernandes , Shirin Razdan , Zachary Barnes , Fanny Collado , Connie Sol , Mary Ann Fletcher and Nancy Klimas, A comparison of sex-specific immune signatures in Gulf War illness and chronic fatigue syndrome, BMC Immunology 14 (2013).
[14] G.K. Broderick, Ben Z; Fernandes, Henrique; Fletcher, Mary Ann; Klimas, Nancy; Smith, Frederick A; O'Gorman, Maurice RG; Vernon, Suzanne D; Taylor, Renee, cytokine expression profiles of immune imbalance in post-mononucleosis chronic fatigue, Journal of Translational Medicine 10 (2012).
[15] A.T. White, A.R. Light, R.W. Hughen, L. Bateman, T.B. Martins, H.R. Hill, K.C. Light, Severity of symptom flare after moderate exercise is linked to cytokine activity in chronic fatigue syndrome, Psychophysiology 47(4) (2010) 615-24.
[16] T. Nakamura, S.K. Schwander, R. Donnelly, F. Ortega, F. Togo, G. Broderick, Y. Yamamoto, N.S. Cherniack, D. Rapoport, B.H. Natelson, Cytokines across the night in chronic fatigue syndrome with and without fibromyalgia, Clinical and vaccine immunology : CVI 17(4) (2010) 582-7.
[17] V.A. Spence, G. Kennedy, J.J. Belch, A. Hill, F. Khan, Low-grade inflammation and arterial wave reflection in patients with chronic fatigue syndrome, Clin Sci (Lond) 114(8) (2008) 561-6.
[18] U. Vollmer-Conna, B. Cameron, D. Hadzi-Pavlovic, K. Singletary, T. Davenport, S. Vernon, W.C. Reeves, I. Hickie, D. Wakefield, A.R. Lloyd, G. Dubbo Infective Outcomes Study, Postinfective fatigue syndrome is not associated with altered cytokine production, Clin Infect Dis 45(6) (2007) 732-5.
[19] K.L. MacDonald, T.O. Michael, K.H. LeDell, K.E. White, C.H. Schenck, C.C. Chao, D.H. Persing, R.C. Johnson, J.M. Barker, P.K. Peterson, A case-Control Study to Assess Possible Triggers and Cofactors in Chronic Fatigue Syndrome, The American Journal of Medicine 100 (1996).
[20] C.M.A. Swanink, J.H.M.M. Vercoulen, J.M.D. Galama, M.T.L. Roos, L. Meyaard, J.v.d. Ven-Jongekrijg, R.d. Nijs, G. Bleijenberg, J.F.M. Fennis, F. Miedema, Lymphocyte subsets, apoptosis, and cytokines in patients with chronic fatigue syndrome, Journal of Infectous Diseases 173(2) (1996).
[21] R. Patarca, N.G. Klimas, S. Lugtendorf, M. Antoni, M.A. Fletcher, Dysregulated Expression of Tumor Necrosis Factor in Chronic Fatigue Syndrome: Interrelations with Cellular Sources and Patterns of Soluble Immune Mediator Expression, Clinical Infectious Diseases 18(Supplement_1) (1994) S147-S153.
[22] A. Lloyd, S. Gandevia, A. Brockman, J. Hales, D. Wakefield, Cytokine Production and Fatigue in Patients with Chronic Fatigue Syndrome and Healthy Control Subjects in Response to Exercise, Clinical Infectious Diseases 18(Supplement_1) (1994) S142-S146.
[23] A. Linde, B. Andersson, S.B. Svenson, H. Ahrne, M. Carlsson, P. Forsberg, H. Hugo, A. Karstorp, R. Lenkei, A. Lindwall, A. Loftenius, C. Säil, J. Andersson, Serum Levels of Lymphokines and Soluble Cellular Receptors in Primary Epstein-Barr Virus Infection and in Patients with Chronic Fatigue Syndrome, The Journal of Infectious Diseases 165(6) (1992) 994-1000.

[24] C.C. Chao, E.N. Janoff, S.X. Hu, K. Thomas, M. Gallagher, M. Tsang, P.K. Peterson, Altered cytokine release in peripheral blood mononuclear cell cultures from patients with the chronic fatigue syndrome, Cytokine 3(4) (1991) 292-8.
[25] S.E. Straus, J.K. Dale, J.B. Peter, C.A. Dinarello, Circulating Lymphokine Levels in the Chronic Fatigue Syndrome, The Journal of Infectious Diseases 160(6) (1989) 1085-1086.
[26] Y. Jammes, J.G. Steinberg, S. Delliaux, F. Bregeon, Chronic fatigue syndrome combines increased exercise-induced oxidative stress and reduced cytokine and Hsp responses, J Intern Med 266(2) (2009) 196-206.
[27] P.D. White, K.E. Nye, A.J. Pinching, T.M. Yap, N. Power, V. Vleck, D.J. Bentley, J.M. Thomas, M. Buckland, J.M. Parkin, Immunological Changes After Both Exercise and Activity in Chronic Fatigue Syndrome, Journal of Chronic Fatigue Syndrome 12(2) (2004) 51-66.
[28] J. Visser, W. Graffelman, B. Blauw, I. Haspels, E. Lentijes, E.R.d. Kloet, L. Nagelkerken, LPS-induced IL-10 production in whole blood cultures from chronic fatigue syndrome patients is increased but supersensitive to inhibition by dexamethasone, Journal of Neuroimmunology 119 (2001).
[29] K. Nas, R. Cevik, S. Batum, A.J. Sarac, S. Acar, S. Kalkanli, Immunologic and psychosocial status in chronic fatigue syndrome, Bratisl Lek Listy 112(4) (2011) 208-12.
[30] M. Robinson, S.R. Gray, M.S. Watson, G. Kennedy, A. Hill, J.J. Belch, M.A. Nimmo, Plasma IL-6, its soluble receptors and F2-isoprostanes at rest and during exercise in chronic fatigue syndrome, Scand J Med Sci Sports 20(2) (2010) 282-90.
[31] U.M. Nater, L.S. Youngblood, J.F. Jones, E.R. Unger, A.H. Miller, W.C. Reeves, C. Heim, Alterations in diurnal salivary cortisol rhythm in a population-based sample of cases with chronic fatigue syndrome, Psychosom Med 70(3) (2008) 298-305.
[32] J.G. Cannon, J.B. Angel, R.W. Ball, L.W. ABAD, L.R. Fagioli, A.L. Kamaroff, Acute Phase Responses and Cytokine Secretion in Chronic Fatigue Syndrome, Journal of Clinical Immunology 19 (1999).
[33] D. Buchwald, M.H. Wener, T. Pearlman, P. Kith, Markers of inflammation and immune activation in chronic fatigue and chronic fatigue syndrome, J Rheumatol 24(2) (1997) 372-6.
[34] P.R. Cheney, S.E. Dorman, D.s. Bell, Interleukin-2 and the Chronic Fatigue Syndrome, Annals of Internal Medicine 110 (1989).
[35] S. Blundell, K.K. Ray, M. Buckland, P.D. White, Chronic fatigue syndrome and circulating cytokines: A systematic review, Brain Behav Immun 50 (2015) 186-195.
